# Supplementary material for: Identification of photoperiod-induced specific miRNAs in the adrenal glands of Sunite sheep (Ovis aries)
Source: Front Vet Sci. 2022 Jul 22;9:888207. doi: 10.3389/fvets.2022.888207 (PMC9354845; doi:10.3389/fvets.2022.888207)
Supplement: Supplementary file 1 [file Data_Sheet_1.ZIP › Supplementary file/miRDeep2 file.zip!/miRDeep2 file/Know_Structure/SPLP42A3/oar-mir-30c.pdf]

miRBase precursor	: oar-mir-30c
Total read count	: 68362
oar-miR-30c read count	: 68275
remaining reads	: 87

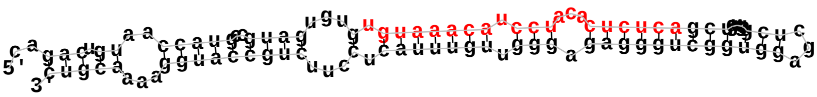

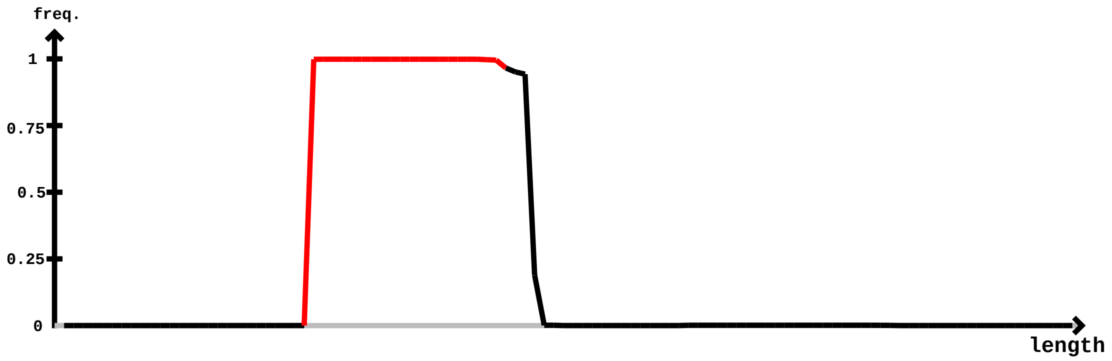


```
                                oar-miR-30c
5'- cagacuguaaccaugccguagugug uguaaacauccuacacucuca gcugugagcucgagguggcugggagaggguuguuuacuccuucugccauggaaaacguc  -3'   exp
    ..(((.((..(((((..((((...(.(((((((.(((...((((((((....(((...)))))))))))).))).))))))).)...)))))))))...)))))   reads  mm  sample
    cagacuguaaccaugccguagugu.................................................................................   2      0   seq
    ....cuguaaccaugccguagugug................................................................................   1      0   seq
    .........................uguaaacauccuacacuU..............................................................   16     1   seq
    .........................uguaaacauccuacacuc..............................................................   95     0   seq
    .........................uguaaacaCccuacacucu.............................................................   14     1   seq
    .........................uguaaacauccuacacucu.............................................................   111    0   seq
    .........................uguaaacauccuacacucuc............................................................   1873   0   seq
    .........................uguaaacaCccuacacucuc............................................................   132    1   seq
    .........................uguaaacaCccuacacucuca...........................................................   51     1   seq
    .........................uguaaacauccuacacucuca...........................................................   977    0   seq
    .........................uguaaacaCccuacacucucag..........................................................   59     1   seq
    .........................uguaaacauccuacacucucag..........................................................   497    0   seq
    .........................uguaaacaCccuacacucucagc.........................................................   2661   1   seq
    .........................uguaaacauccuacacucucagc.........................................................   48848  0   seq
    .........................uguaaacauccuacacucucagcu........................................................   12215  0   seq
    .........................uguaaacaCccuacacucucagcu........................................................   616    1   seq
    .........................uguaaacauccuacacucucagcug.......................................................   6      0   seq
    .........................uguaaacauccuacacucucagcugu......................................................   78     0   seq
    .........................uguaaacaCccuacacucucagcugu......................................................   1      1   seq
    ..........................guaaacauccuacacucuc............................................................   1      0   seq
    ..........................guaaacauccuacacucucagc.........................................................   18     0   seq
    ..........................guaaacaCccuacacucucagc.........................................................   1      1   seq
    ..........................guaaacaCccuacacucucagcu........................................................   1      1   seq
    ..........................guaaacauccuacacucucagcu........................................................   4      0   seq
    ................................................................cugggagaggguuguuuacu.....................   17     0   seq
    ................................................................cugggagaggguuguuuacuc....................   14     0   seq
    ................................................................cugggagaggguuguuuacucc...................   42     0   seq
    .................................................................ugggagaggguuguuuacu.....................   2      0   seq
    .................................................................ugggagaggguuguuuacuc....................   2      0   seq
    .................................................................ugggagaggguuguuuacucc...................   6      0   seq
    .................................................................ugggagaggguuguuuacuccu..................   1      0   seq
```
